# Congenitally Acquired Persistent Lymphocytic Choriomeningitis Viral Infection Reduces Neuronal Progenitor Pools in the Adult Hippocampus and Subventricular Zone

**DOI:** 10.1371/journal.pone.0096442

**Published:** 2014-05-06

**Authors:** Tony Sun, Michael J. Vasek, Robyn S. Klein

**Affiliations:** 1 Department of Internal Medicine, Washington University School of Medicine, Saint Louis, Missouri, United States of America; 2 Department of Anatomy and Neurobiology, Washington University School of Medicine, Saint Louis, Missouri, United States of America; 3 Department of Pathology and Immunology, Washington University School of Medicine, Saint Louis, Missouri, United States of America; University of Utah, United States of America

## Abstract

Lymphocytic choriomeningitis virus (LCMV) can be transmitted through congenital infection, leading to persistent infection of numerous organ systems including the central nervous system (CNS). Adult mice persistently infected with LCMV (LCMV-cgPi mice) exhibit learning deficits, such as poor performance in spatial discrimination tests. Given that deficits in spatial learning have been linked to defects in adult neurogenesis, we investigated the impact of congenital LCMV infection on generation of neuroblasts from neural progenitor cells within neurogenic zones of adult mice. In LCMV-cgPi mice, QPCR and immunohistochemistry detected presence of LCMV glycoprotein-coding RNA and nucleoprotein in the hippocampal dentate gyrus and subventricular zone (SVZ), sites of neurogenesis that harbor populations of neuroblasts. Numbers of neuroblasts were reduced in LCMV-cgPi mice, as determined by IHC quantification, and analysis of BrdU incorporation by flow cytometry revealed lower numbers of BrdU-labeled neuroblasts. Additionally, TUNEL assays performed *in situ* showed increased numbers of apoptotic cells in the two neurogenic regions. Next, neurosphere cultures were infected *in vitro* with LCMV and differentiated to create a population of cells that consisted of both transit amplifying cells and neuroblasts. Immunocytochemical and TUNEL assays revealed increased numbers of TUNEL-positive cells that express nestin, suggesting that the drop in numbers of neuroblasts was due to a combination of impaired proliferation and apoptosis of progenitor cells. LCMV-cgPi mice exhibited transcriptional up-regulation several cytokines and chemokines, including gamma-interferon inducible chemokines CXCL9 and CXCL10. Chronic up-regulation of these chemokines can facilitate a pro-inflammatory niche that may contribute to defects in neurogenesis.

## Introduction

Lymphocytic choriomeningitis virus (LCMV) is an RNA virus that can infect the central nervous system (CNS) during neonatal development or adulthood. LCMV infection can result in various pathologies of the CNS depending on the nature of host-pathogen interactions following infection. In immunocompromised individuals, LCMV can enter the CNS and induce an LCMV-specific cell-mediated pathological inflammation of the meninges and choroid plexus [1]–[7]. Conversely, most immunocompetent individuals clear LCMV prior to infection of the CNS and thus do not typically show signs of CNS pathology [2], [6]–[8]. When mice congenitally acquire LCMV *in utero* or soon after birth (LCMV-cgPi mice), they develop immunological tolerance to LCMV antigens, leading to persistent infection [1]–[4], [6]–[8]. Thus, while acute LCMV infection can present a danger to the CNS in the fetus, child, and adult [9]–[12], it is also possible that congenital LCMV infection can lead to chronic, low-level infections that involve the CNS [13]–[17].

Adult LCMV-cgPi mice exhibit lifelong viremia [14], [18], [19]and shedding of virus in urine and saliva, sustaining exposure of neonatal mice to LCMV antigens. Studies in LCMV-cgPi mice demonstrate that LCMV can infect mitotically active neural progenitor cells [1], [3], [5], [20], [21], leading to up-regulation of pro-inflammatory cytokines such as IL-1 [8], [22], [23], reduced numbers of hippocampal dentate granule cells [24]–[26], and congenital defects in the brain such as microcephaly and hydrocephalus [20], [21], [27]. In the brain, LCMV-infected neurons are not lysed as a result of the virus being present [9]–[12], [25], and this non-cytopathic feature enables LCMV to replicate and persist in the CNS. Many neurons are infected in the cerebral cortex, limbic system structures, cerebellum, and hippocampus, and infected brain regions show up-regulation of interferon-stimulated genes, including the transcription factors STAT1 and IRF9 [14], [16], [17], [21]. It has been suggested that chronic up-regulation of interferons or alternations in adult neurogenesis may contribute to spatial learning deficits observed in LCMV-cgPi mice [9]–[12], [14], [19] [1], [3], [5], [14], [16], [17], [21], however, the long-term effects of LCMV infection on adult neuronal physiology and neurogenesis remain relatively unexplored. The process of neurogenesis occurs in the subgranular zone (SGZ) of the hippocampal dentate gyrus [8], [14], [19], [23] and the subventricular zone (SVZ) of the lateral ventricles [14], [28], [29]. Under normal conditions, neural progenitor cells in the SGZ differentiate and join the granule cell layer of the dentate gyrus, while the same cell types in the SVZ eventually differentiate into olfactory bulb neurons. The homeostasis of these neurogenic regions during persistent LCMV infection remains largely unknown. However, correlations have been observed between neurologic sequelae and congenital LCMV infection, leading to the broad hypothesis that *in utero* viral infections contribute to the development of idiopathic CNS disorders [10], [14], [30].

In the present study, the impact of chronic LCMV infection on neural progenitor cells was investigated following vertical viral transmission, which established persistent infection in the SVZ and hippocampus. LCMV antigen was detected in both neuroblasts within the SVZ and SGZ, and in neurons within the olfactory bulb and granule cell layer of the hippocampus. In neurogenic regions of LCMV-cgPi mice, there were fewer total and BrdU-labeled neuroblasts. In *in vitro* studies, LCMV infection induced apoptosis of Nestin^+^ neural progenitors, but did not affect the lineage fate of surviving cells. The *in vivo* reduction in the neural progenitor pool resulted in lower numbers of neuroblasts, which are direct precursors to mature neurons. LCMV-infected SVZ and hippocampal tissue also showed significant upregulation of gamma-interferon (IFN-γ) inducible chemokines CXCL9 and CXCL10. These data suggest that congenital infection with LCMV may lead to depletion of neural progenitors and persistent expression of inflammatory chemokines.

## Materials and Methods

### Animals and antibodies

Wild-type C57BL/6 mice were obtained from Jackson Labs, Bar Harbor, ME and kept under pathogen free conditions (Department of Comparative Medicine, Washington University, St. Louis, MO). Experiments with mice were performed under approved guidelines as outlined by the Washington University School of Medicine Animal Studies Committee. Purified mouse monoclonal anti-LCMV-nucleoprotein antibody (1.1.3) from hybridoma supernatant was provided by Dr. J. Carlos de la Torre (The Scripps Institute, La Jolla, CA). Sheep anti-BrdU, chicken polyclonal anti-beta III tubulin, and goat polyclonal anti-IBA1 antibodies were purchased from Abcam. Rabbit anti-doublecortin (DCX) polyclonal antibody was purchased from Cell Signaling. Rabbit anti-nestin polyclonal antibody was purchased from Millipore. Fluorescently-conjugated antibodies against CD45.2 (fluoroscein isothiocyanate, FITC) was purchased from BD Pharmingen. Rat monoclonal anti-GFAP antibody and fluorescently-conjugated secondary antibodies were purchased from Invitrogen. Normal goat and donkey sera and isotype controls were purchased from Jackson ImmunoResearch Laboratories (West Grove, PA).

### Mouse model (LCMV-cgPi mice)

LCMV infection was established by intracranial inoculation of neonatal C57BL/6 wild-type mice with 3×10^5^ pfu of LCMV-Armstrong strain 53b [13]–[15], [17]. LCMV stocks were grown and expanded in BHK-21 cells, and viral titers were determined by plaque assay on Vero cell monolayers [18], [31]. Positive infection was determined by presence of LCMV RNA in serum as detected by QPCR, and these infected mice were used as breeders to establish a colony of LCMV-cgPi mice through mother-to-offspring transmission of virus. LCMV-cgPi mice were kept in BSL-2 facilities [20], [32], [33]. All experiments were performed with age-matched uninfected controls at 6 weeks of age.

### SVZ and hippocampal tissue extraction

Mice were perfused intracardically with ice-cold 0.01 M (1X) PBS solution (Sigma-Aldrich), and tissue samples were harvested for QPCR and flow cytometry. To do this, whole brain was harvested and kept on ice-cold Hibernate-A (Gibco) for manual dissection of the hippocampus and subventricular zone under a stereomicroscope [22], [27]. Tissue samples harvested for QPCR were placed in TRI Reagent (Ambion) and set on dry ice at −80°C. For flow cytometry, dissected tissue samples were placed in Hibernate-A and kept on ice at 4°C.

### Flow cytometry

To quantify BrdU-labeled doublecortin (DCX)-positive neuroblasts, BrdU was diluted in sterile 1× PBS and injected intraperitoneally at 100 mg/kg in 24 hr intervals 2 days before sacrifice. To isolate neural progenitor cells for flow cytometry, tissue samples were triturated in HBSS (Invitrogen), followed by enzymatic dissociation in 0.25% trypsin (Gibco) with bovine DNase-I (Sigma-Aldrich) in a 37°C water bath for 10 min. Samples were then filtered through a 70 µm cell strainer and centrifuged for 20 min in 30% Percoll solution (Sigma-Aldrich) diluted in 1× PBS. The centrifugation separated out myelin and other cellular debris, which were subsequently removed with the Percoll solution. Pelleted cells were separated into sample tubes and pooled together for compensation controls. Each sample was incubated in Fc receptor block, fixed, and stained for surface antigens, including CD45. Next, cells were permeabilized and stained for intracellular antigens (DCX, BrdU). BrdU staining was done using the FITC BrdU flow kit (BD Biosciences). Finally, fluorescently-conjugated secondary antibodies and DAPI were added to samples before quantification on the LSR II Flow Cytometer (BD Biosciences).

### Quantitative immunohistochemistry

To quantify BrdU-labeled doublecortin-positive neuroblasts, one dose of BrdU was injected intraperitoneally at 150 mg BrdU/kg body weight 48 hr before sacrifice. Whole brain was isolated after perfusion with ice-cold PBS 4% paraformaldehyde (PFA) solution. Whole brain was then fixed overnight in 4% PFA on ice at 4°C. After the overnight fixation, tissue samples were incubated for 24 hr in 4°C in a 30% sucrose solution. Whole brain was split to separate the left and right hemispheres, and each hemisphere was cut coronally. 8 µm frozen sections were washed in PBS, permeabilized with 0.1% Triton X-100 (Sigma-Aldrich), and blocked with 5% normal goat serum for 1 hr at room temperature. Monoclonal or polyclonal antibodies were diluted to 1–10 g/ml in PBS containing 0.1% Triton X-100 and 5% normal goat serum. Primary antibodies and isotype controls were applied overnight at 4°C. Primary antibodies were detected by secondary antibodies conjugated to Alexa Fluor 488 or Alexa Fluor 555 (Invitrogen) for visualization on an epi-fluorescent Zeiss microscope. Nuclei were counterstained with DAPI. Apoptosis was assessed using TUNEL assays (Roche).

### Real-time QPCR

Total RNA was isolated from whole tissue, and QPCR runs were performed as previously described [24], [26], [34]. Total RNA copies were normalized to copies of GAPDH. Primer sequences used to amplify GAPDH, CXCL9, CXCL10, CCL2, CCL5, CCL19, CCL21, IFN-γ, TNF-α, and LCMV-glycoprotein were previously published [20], [27], [35]. The primer sequences for IFN-α and IFN-β, respectively, are F: 5′ CTT CCA CAG GAT CAC TGT GTA CCT 3′, R: 5′ TTC TGC TCT GAC CAC CTC CC 3′ and F: 5′ CTG GAG CAG CTG AAT GGA AAG 3′, R: 5′ CTT CTC CGT CAT CTC CAT AGG G 3′.

### Neurosphere cell culture

To prepare a neurosphere culture, the subventricular zone was dissected from neonatal mice and enzymatically dissociated with 0.25% trypsin and bovine DNase-I. Cells were passed through a 70 µm cell strainer followed by a 40 µm cell strainer and then plated with proliferation media consisting of neurosphere basal media (low-glucose DMEM, F12, HEPES, penicillin/streptomycin) supplemented with B27, mouse EGF, and human FGF2 (Invitrogen). To induce differentiation of neurospheres, growth factors were removed and N2 was added to the neurosphere basal media along with B27. Neurospheres were re-fed every 48 hr and used for up to 12 passages.

To infect neurospheres with LCMV, the cells were first plated in 24-well plates at a density of 125,000 cells/well on poly-d-lysine/laminin-coated coverslips (Invitrogen). The virus was diluted in neurosphere basal media and used to infect cells at 1 M.O.I. Infected cell cultures were incubated in a BSL-II primary tissue culture incubator at 37°C with 5% CO_2_. To perform quantitative immunocytochemistry, cell cultures were fixed for 15 min at room temperature in 4% PFA and incubated in primary antibodies for 1 hr at room temperature. Secondary antibodies were then added for 1 hr at room temperature.

### Statistical testing

All statistical analyses were performed with software: GraphPad Prism 5.0. Numerical values on graphs were expressed as mean ± standard error of mean (SEM). Student's t-tests were performed in two-group comparisons, and differences were defined to be statistically significant when p<0.05.

## Results

### LCMV infects the subventricular zone and hippocampus

**Figure 1:**
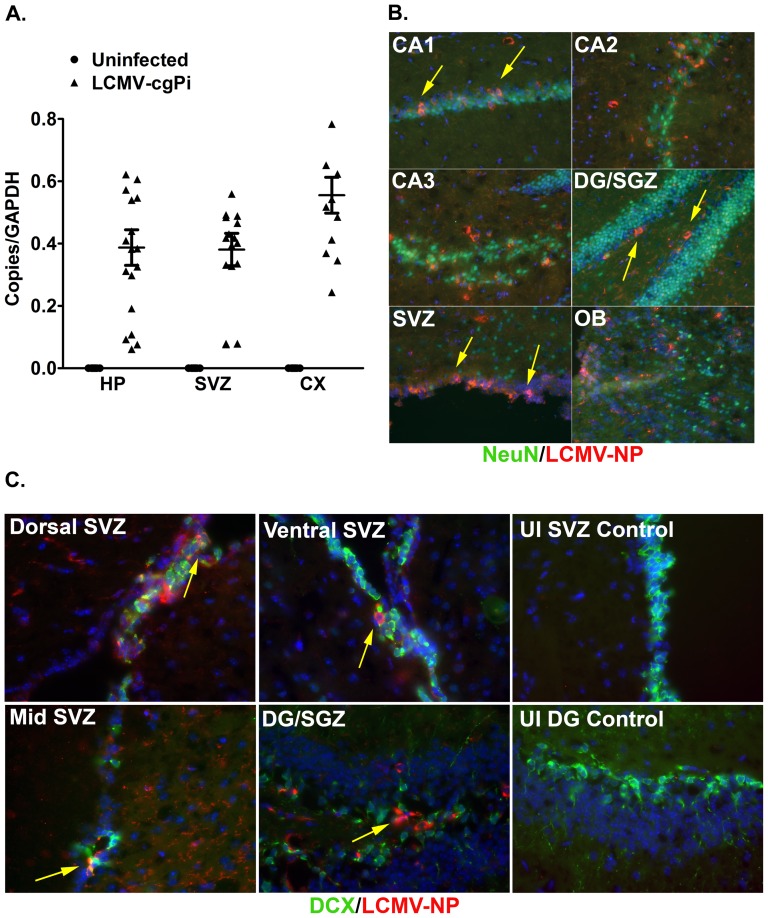
LCMV infects cells in the hippocampus and subventricular zone. In 6-week old LCMV-cgPi mice, (**A**) RT-QPCR quantification of LCMV-glycoprotein RNA in hippocampus (HP), subventricular zone (SVZ), and cortex (CX) is shown with mean ± SEM; N≥10 mice. Uninfected controls were included in the experiment to demonstrate primer specificity. (**B**) Representative IHC images show infection of neurons (NeuN^+^ cells) in the SVZ, olfactory bulb (OB), and regions of hippocampus: subgranular zone (SGZ) of dentate gyrus (DG) and CA1, CA2, and CA3. Tissue sections were stained with anti-LCMV-nucleoprotein and anti-NeuN. Table 1 shows quantitative data for levels of infection. (**C**) Representative IHC images show infection of neuroblasts (DCX^+^ cells) in the SVZ and SGZ. Tissue sections were stained with anti-LCMV-nucleoprotein (NP) and anti-DCX. Uninfected controls were included for all IHC experiments that used the anti-LCMV-nucleoprotein antibody.

Previous studies have shown that LCMV-cgPi mice have spatial learning deficits and viral antigen present in the cortex, cerebellum, and hippocampus [14], [28]. In the current study, we examined the impact of persistent LCMV infection on homeostasis in the SVZ and hippocampus. Total RNA from manually dissected hippocampal and SVZ tissue revealed the presence of LCMV glycoprotein-coding RNA in both neurogenic regions, as assayed by quantitative reverse-transcriptase (Q)PCR. The relative levels of viral RNA ranged between 0.05 and 0.5 copies per copy of GAPDH, suggesting single log-fold variation in viral replication within the hippocampus and SVZ (**Figure 1A**). The variation in levels of LCMV in 6 week old LCMV-cgPi mice is consistent with observations in 3–6 month old LCMV-cgPi mice [14], [36]. Tissue samples from infected cortex were used as positive controls for RT-QPCR detection of LCMV RNA [14], [17], [37]. In the positive controls, LCMV RNA levels ranged between 0.5 and 1.0 copies.

Immunohistochemistry was performed to localize LCMV infection in the hippocampus and SVZ (N = 6 mice). In the hippocampus, infected NeuN^+^ neurons were present in the cornus ammonis (CA) regions (Figure 1B, top, mid left), dentate gyrus (DG), and subgranular zone (SGZ) (**Figure 1B** **, mid right**). LCMV-infected neurons were also detected in the olfactory bulb (**Figure 1B** **, bottom right**). As expected for the SVZ, very few neurons were observed to line the lateral walls of the ventricles, but other cell types present there were infected with LCMV (**Figure 1B** **, bottom left**). LCMV nucleoprotein staining was also co-localized with doublecortin (DCX) staining to detect LCMV-infected neuroblasts within the SVZ and SGZ (**Figure 1C**). Quantitation of LCMV^+^NeuN^+^ neurons (N = 6 mice) within olfactory bulb and hippocampal subregions revealed they comprised 5–30% of total NeuN^+^ neurons while infected neuroblasts comprise 49% and 45% of total infected cells in the SGZ and SVZ (**Table 1**).

**Table 1:** Distribution of LCMV in neurons within the olfactory bulb (OB), dentate gyrus (DG), CA1, CA2, and CA3 (+, <10%; ++, 10–30%; +++, 30–50%).

	OB	DG	CA1	CA2	CA3
Animal 1	+++	+	++	++	+++
Animal 2	++	+	+	++	++
Animal 3	+++	++	++	++	+++
Animal 4	+++	+	+++	+++	+++
Animal 5	+++	+	++	++	+++
Animal 6	+++	+	++	+++	+++

### Numbers of neuroblasts are decreased in LCMV-cgPi mice

**Figure 2:**
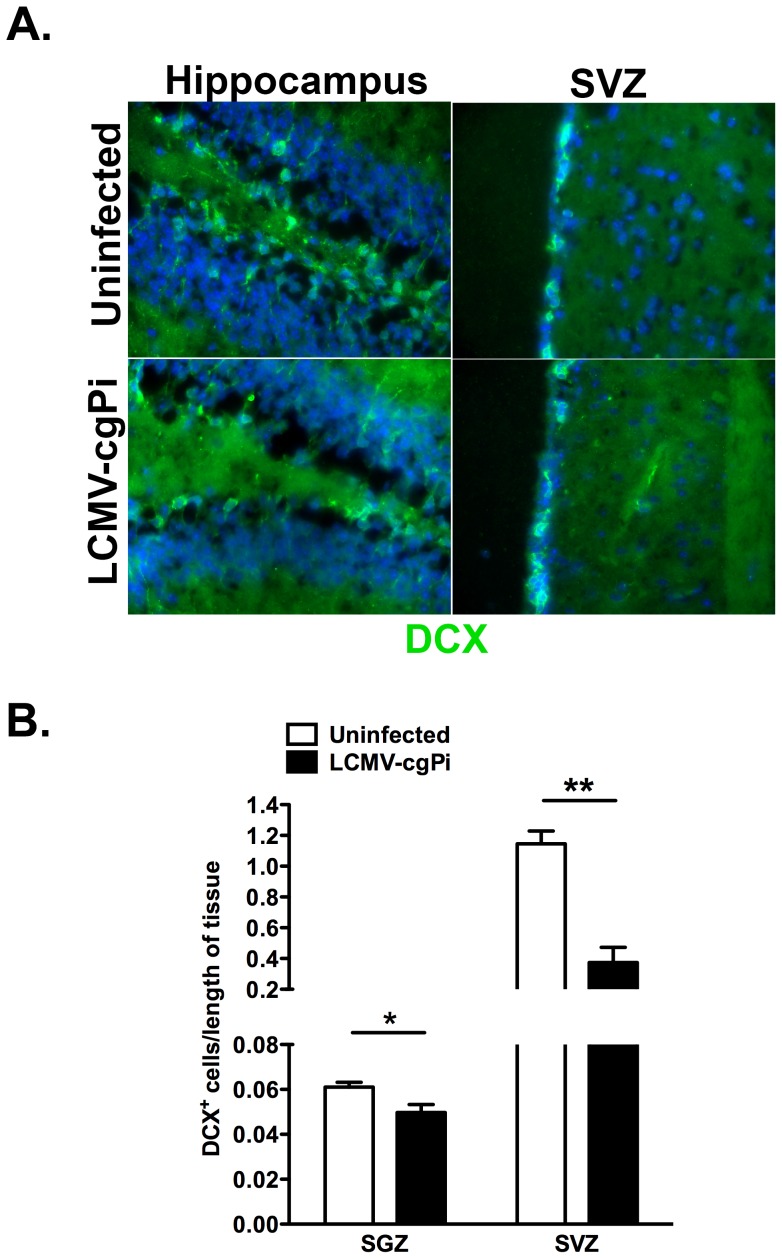
Numbers of neuroblasts are decreased in LCMV-cgPi mice. In age-matched 6-week old mice, (**A**) representative IHC images show a comparison of neuroblast numbers between uninfected mice and LCMV-cgPi mice. (**B**) Quantitative IHC analysis revealed lower numbers of neuroblasts in LCMV-cgPi mice. Numbers of neuroblasts were normalized to the lengths of the SGZ and SVZ. Data points are shown with mean ± SEM, N = 4 mice; *p<0.05, **p<0.01.

Following detection of LCMV glycoprotein-coding RNA and LCMV nucleoprotein in both the SGZ of the dentate gyrus and the SVZ of the lateral ventricles, we investigated how LCMV persistence in the CNS impacts on numbers and proliferation of neural progenitor cells, which differentiate and reduce mitotic activity to form DCX-expressing neuroblasts [27], [31]. Quantitative immunohistochemistry for DCX along the SGZ of the dentate gyrus and SVZ revealed decreased total numbers of DCX^+^ neuroblasts (**Figure 2**).

To investigate the proliferation of neural progenitor cells in these two neurogenic regions, we performed BrdU labeling studies in which two pulses of BrdU in 24 hour intervals at 48 hours prior to sacrifice to track neural progenitor cell proliferation using flow cytometry. The percentage of BrdU^+^DCX^+^ neuroblasts was used as the quantitative readout of both neuroblast and neural progenitor cell proliferation. We selected the population of neuroblasts by gating on live cells and singlets, followed by CD45^−^ cells, and then DCX^+^ neuroblasts. Finally, the number of BrdU-labeled neuroblasts was normalized to total numbers of neuroblasts.

**Figure 3:**
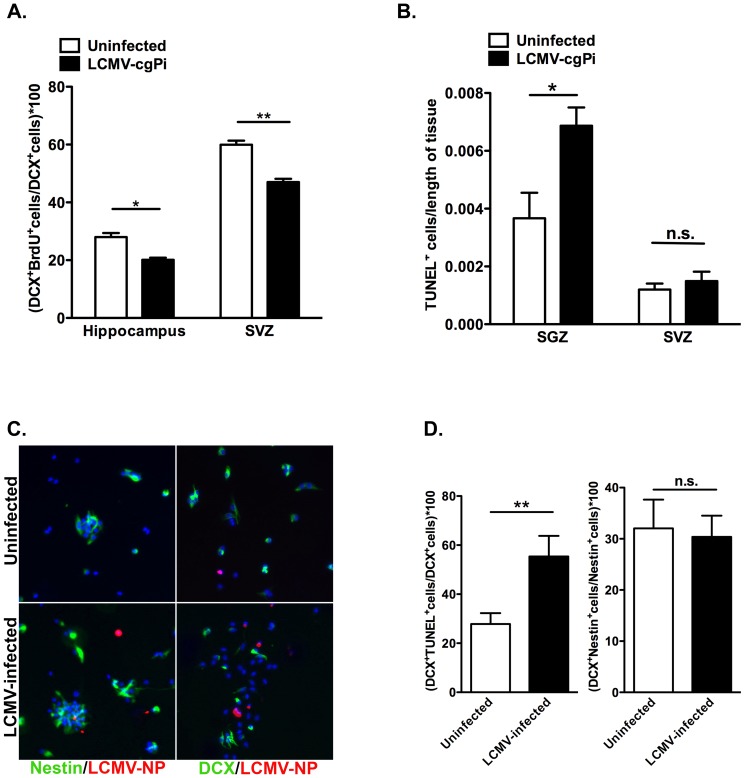
Proliferation and survival of neural progenitor cells are affected in LCMV-cgPi mice. In age-matched 6-week old mice, (**A**) analysis of neural progenitor cells using flow cytometry revealed lower percentages of BrdU-labeled neuroblasts in the hippocampus and SVZ of LCMV-cgPi mice. Data points are shown with mean ± SEM, N≥4 mice; *p<0.05, **p<0.01. (**B**) Quantitative IHC analysis revealed greater numbers of TUNEL^+^ cells in the SGZ of LCMV-cgPi mice. Data points are shown with mean ± SEM, N = 3 mice. (**C**) Neurospheres were infected with LCMV, and growth factors were removed to induce differentiation. Representative ICC images show Nestin^+^ transit amplifying cells and DCX^+^ neuroblasts in neurosphere cultures at 12 hours following withdrawal of growth factors. (**D**) Quantitative ICC analysis revealed increased numbers of TUNEL^+^Nestin^+^ cells in the LCMV-infected cell cultures. Data points are shown with mean ± SEM, N = 3 wells.

Compared with uninfected mice, LCMV-cgPi mice showed a significant reduction in numbers of BrdU-labeled DCX^+^ neuroblasts in the SVZ and hippocampus, suggesting reduced neurogenesis (**Figure 3A**). The percentage of BrdU^+^DCX^+^/total DCX^+^ is a measure of the generation of neuroblasts through both proliferation and differentiation of neural progenitor cells and proliferation in the existing neuroblast population. In the SVZ, uninfected mice showed 60% BrdU^+^DCX^+^/total DCX^+^ while LCMV-cgPi mice showed 47% BrdU^+^DCX^+^/total DCX^+^ (p<0.01). In the hippocampus, uninfected mice showed 28% BrdU^+^DCX^+^/total DCX^+^ compared to 20% for LCMV-cgPi mice (p<0.05).

Whole brains from LCMV-cgPi mice and uninfected mice were sectioned and stained with a TUNEL kit to look at cell survival. Quantitative immunohistochemistry was performed to quantify TUNEL^+^ cells along the SGZ of the dentate gyrus and SVZ. To get the total number of TUNEL^+^ cells in the SGZ, numbers of TUNEL^+^ cells were added up from the groove of the dentate gyrus through the SGZ on both arms of the dentate gyrus. For the SVZ, numbers of TUNEL^+^ cells were added from the top to the bottom of the lateral ventricle walls, as observed in coronal sections. Total numbers of TUNEL^+^ cells were then normalized to the lengths of the SGZ of the dentate gyrus and the lateral ventricles walls, much like what was done to determine neuroblast numbers. Each data point represents one animal and includes the average of cell counts from five slides. LCMV-cgPi mice had increased numbers of TUNEL^+^ cells (**Figure 3B**).

To determine the apoptotic cell identities, neurosphere cultures were prepared from whole SVZ tissue. Growth factors were withdrawn to induce differentiation, and cultures were fixed after 12 hours for immunocytochemistry to stain for DCX and Nestin, a marker of neural progenitor cells (**Figure 3C**). Quantitative staining revealed increased numbers of TUNEL^+^Nestin^+^ cells in LCMV-infected cultures (**Figure 3D**). When growth factors were withdrawn for 72 hours to assess effects of LCMV infection on neurosphere differentiation patterns, no change was observed in numbers of differentiated GFAP^+^ astrocytes or beta tubulin III^+^ neurons, as observed by quantitative immunocytochemistry (**Figure S1**).

### Chemokine signaling is affected in the neurogenic regions of LCMV-cgPi mice

**Figure 4:**
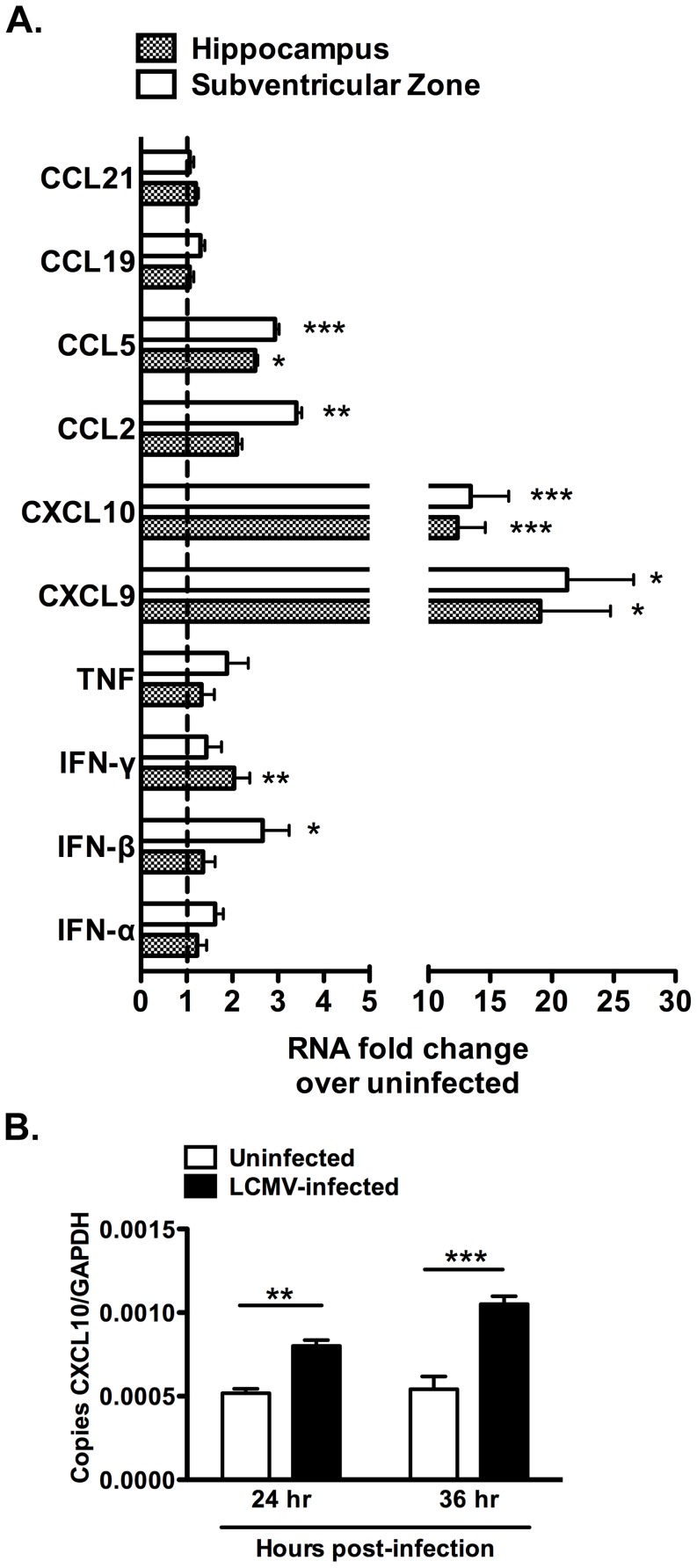
Chemokine signaling is affected in the neurogenic regions of LCMV-cgPi mice. In age-matched 6-week old mice, (**A**) RT-QPCR analysis revealed transcriptional upregulation of CXCL9, CXCL10, and CCL5 in the hippocampus and SVZ; CCL2 in the SVZ; IFN-γ in the hippocampus; and IFN-β in the SVZ. CCR7 ligands, CCL19 and CCL21, IFN-α, and TNF were not significantly upregulated. Data points are shown with mean ± SEM, N≥10 mice; *p<0.05, **p<0.01, ***p<0.001. (**B**) RT-QPCR analysis of RNA extracted from neurospheres revealed transcriptional upregulation of CXCL10 in LCMV-infected cell cultures. Other chemokines and cytokines were not significantly upregulated. Data points are shown with mean ± SEM, N = 6 wells.

Previous studies have provided evidence that implicate chemokines as important regulatory and signaling molecules in the regulation of neurogenesis [32], [33], [35]. Chemokines, such as CXCL10, are also produced by neurons in response to viral infections, such as West Nile viral infections [27], [35]. Other chemokines may be upregulated in the SVZ and hippocampus of LCMV-cgPi mice, and characterizing the expression of these signaling and chemoattractant molecules may be important to understanding why neurogenesis is altered during persistent LCMV infection. Using real-time QPCR, we observed transcriptional up-regulation of several chemokines. In the group of chemokines and cytokines analyzed, two gamma-interferon (IFN-γ)-inducible chemokines: CXCL9 and CXCL10 showed the greatest fold change in expression in LCMV-cgPi mice. CXCL9 was induced by nearly twice log-fold, p<0.05, while CXCL10 expression was induced by about one log-fold, p<0.001 (**Figure 4A**). Similar results were observed for CXCL10 in vitro for LCMV-infected neurospheres (**Figure 4B**).

Expression of Type I and II interferons was also observed to be upregulated between 1–3 fold in LCMV-cgPi mice. The lower induction of Type I and II interfereons is consistent with the previously described inhibition of type I interferon antiviral responses by arenavirus nucleoprotein-mediated interference of transcription factor activation by cytoplasmic pattern recognition receptors [34], [38], [39]. Two CCR5 ligands, CCL2 and CCL5, were also upregulated. The CCR7 ligands, CCL19 and CCL21, were not observed to be upregulated. The precise functions of these chemokines in regulating neurogenesis *in vivo* have been incompletely described, but they have been shown to affect proliferation and differentiation *in vitro* [35], [40].

Though the chemokine environment was altered in a manner consistent with a low-level inflammatory niche, there was no change in numbers of GFAP^+^ astrocytes or IBA^+^ activated microglia, as observed by quantitative IHC (**Figure S2**).

## Discussion

In the current study, the effects of persistent LCMV infection on neurogenesis were investigated in LCMV-cgPi mice, which sustained LCMV infection in neuroblasts and neurons in the hippocampus and SVZ, sites of active neurogenesis. Previously, LCMV-cgPi mice have been observed to exhibit spatial learning deficits [14], [41], a phenotype that suggests defects in neurogenesis, which has been implicated in learning and memory. Though they show behavioral abnormalities, previously published data indicated that the LCMV-cgPi mice and sex- and age-matched controls have similar gross appearances, body weights, and blood glucose levels, suggesting no overt clinical abnormalities [14].

However, LCMV-cgPi mice were observed to have reduced total numbers of neuroblasts in the SVZ and SGZ. Further analysis with BrdU and TUNEL assays suggested that reduced pool of neuroblasts was due to a combination of reduced proliferation of neuroblasts and increased apoptosis of neural progenitor cells. Moreover, reduced numbers of BrdU^+^DCX^+^ cells suggests reduced proliferation in the neural progenitor pool in addition to reduced proliferation in the neuroblast pool, because some of the BrdU incorporation would have occurred when the neuroblasts were still neural progenitor cells.

LCMV-cgPi mice also exhibited significant transcriptional up-regulation of IFN-γ inducible chemokines, CXCL9 and CXCL10, as well as CCL2 and CCL5. CCL2 has been implicated in stroke models to promote the motility of adult neural progenitor cells and enhancing their differentiation into neurons [14], [28], [36]. CCL2 is also expressed in the ventral midbrain and is thought to play a role in development of the CNS [37]. Neuronal CXCL10 production has been shown to play a role in recruiting leukocytes to clear viral infections in the brain, such as during WNV encephalitis [27]. The effects of these chemokines on neurogenesis during persistent LCMV infection still need to be elucidated.

In LCMV-cgPi mice, flow cytometry and quantitative immunohistochemistry showed reduced proliferation of neural progenitor cells in the hippocampus and SVZ. Various molecular mechanisms may account for this impairment in neurogenesis and further studies using chemokine receptor-deficient LCMV-cgPi mice are required. Numerous cytokines and chemokines have been implicated as candidates for regulation of neural progenitor cell proliferation and differentiation, including IFN-γ, CXCL9, CXCL10, CCL2, CCL21, and CXCL12 [32], [35]. CXCL9, which can be induced by IFN-γ, has been implicated to promote neuronal differentiation and inhibit proliferation *in vitro* [35]. CXCL10 displays neurotoxic effects in various infectious diseases, including mediating demyelination during JHMV infection and inducing neuronal apoptosis during chronic, HIV-1 associated neurological disorders [38], [39]. The current paradigm relating inflammation to neurogenesis associates pro-inflammatory molecules with inhibition of neurogenesis and anti-inflammatory molecules with stimulation of neurogenesis. Pro-inflammatory molecules such as IFN-γ and IL-1 are released from activated microglia and inhibit neurogenesis, whereas anti-inflammatory molecules such as IL-4 promote neurogenesis by inducing microglia to secrete stimulatory factors such as IGF-1 [40]. Further studies using knockout mice or RNAi technology are necessary to elucidate the roles of chemokine signaling pathways in regulating neurogenesis in LCMV-cgPi mice.

Importantly, perturbation of neurogenesis during viral infections of the CNS may be linked to various idiopathic CNS disorders. Several studies have established the correlation between neurobehavioral disorders and viral infections of the CNS, including HSV-1, hepatitis C virus, HIV-1, influenza virus, cytomegalovirus, and LCMV infections [41]. Different models of LCMV infection, including vertical transmission and intracranial infection, have been employed to explore the potential link between neurological deficits and persistent LCMV infection. Deficits in learning and memory have been correlated with inflammation, induction of interferon stimulated genes, and altered CNS gene expression [14], [28]. Defects in neurogenesis also occur during persistent LCMV infection, and this may also be linked to cognitive deficits. It is likely that a combination of the previously mentioned factors contributes to problems in learning and memory, and knowledge of the pathways involved in neurogenesis and CNS inflammation can help in developing treatments.

## Supporting Information

Figure S1 **LCMV infection does not affect neural progenitor cell differentiation patterns**. (**A**) 72 hours after withdrawal of growth factors, representative ICC images show beta-tubulin III^+^ neurons and GFAP^+^ astrocytes. (**B**) Quantitative ICC revealed no significant changes in numbers of neurons or astrocytes in the LCMV-infected cell culture. Data points are shown with mean ± SEM, N = 3 wells.
(TIF)

Figure S2 **Numbers of activated microglia are not significantly increased in LCMV-cgPi mice**. In age-matched 6-week old mice, (**A**) **and** (**B**) quantitative IHC analysis revealed no significant differences in total numbers of IBA1^+^ cells (activated microglia) and GFAP^+^ cells (astrocytes) in the SGZ and SVZ. Data points are shown with mean ± SEM, N = 3 mice.
(TIF)
