# Supplementary figures and images for: Congenitally Acquired Persistent Lymphocytic Choriomeningitis Viral Infection Reduces Neuronal Progenitor Pools in the Adult Hippocampus and Subventricular Zone
Source: PLoS One. 2014 May 6;9(5):e96442. doi: 10.1371/journal.pone.0096442 (PMC4011784; doi:10.1371/journal.pone.0096442)

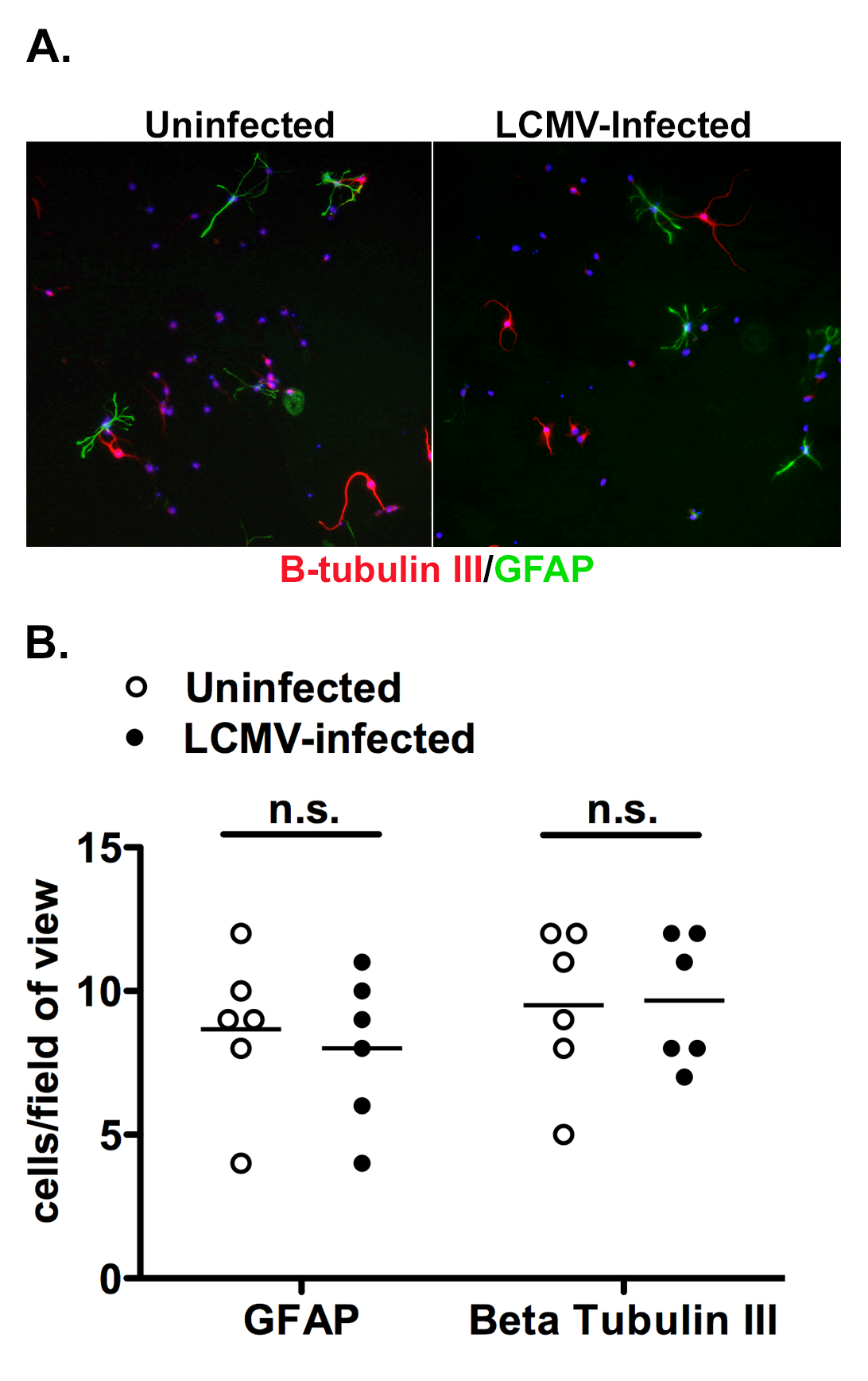

Supplement: Figure S1 — LCMV infection does not affect neural progenitor cell differentiation patterns. (A) 72 hours after withdrawal of growth factors, representative ICC images show beta-tubulin III+ neurons and GFAP+ astrocytes. (B) Quantitative ICC revealed no significant changes in numbers of neurons or astrocytes in the LCMV-infected cell culture. Data points are shown with mean ± SEM, N = 3 wells. (TIF) [file pone.0096442.s001.tif]

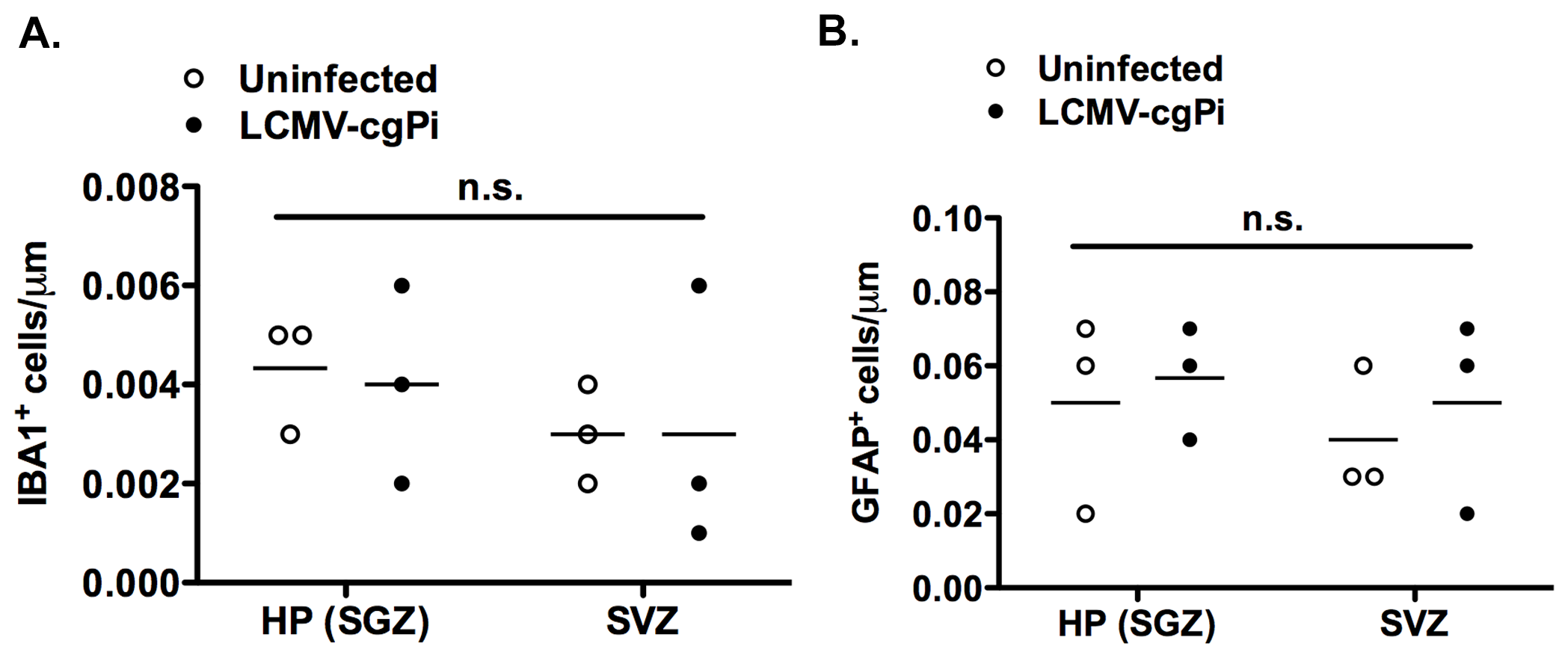

Supplement: Figure S2 — Numbers of activated microglia are not significantly increased in LCMV-cgPi mice. In age-matched 6-week old mice, (A) and (B) quantitative IHC analysis revealed no significant differences in total numbers of IBA1+ cells (activated microglia) and GFAP+ cells (astrocytes) in the SGZ and SVZ. Data points are shown with mean ± SEM, N = 3 mice. (TIF) [file pone.0096442.s002.tif]
